# NIR-II Responsive Platinum-Engineered Vanadium Carbide MXene Endows Poly-L-Lactic Acid Bone Scaffold with Photothermal Antibacterial Property

**DOI:** 10.3390/polym18030378

**Published:** 2026-01-30

**Authors:** Lin Sun, Zihao Zhang, Bingxin Sun, Zhiheng Yu, Guoyong Wang

**Affiliations:** 1 Jiangxi Provincial Key Laboratory of Additive Manufacturing of Implantable Medical Device, Jiangxi University of Science and Technology, Nanchang 330013, China; sunlin2026@163.com (L.S.); 6720231542@mail.jxust.edu.cn (Z.Z.);; 2 College of Mechanical and Electrical Engineering, Jiaxing Nanhu University, Jiaxing 314001, China

**Keywords:** infectious bone defects, vanadium carbide, platinum, photothermal therapy, antibacterial scaffold

## Abstract

Vanadium carbide (V_2_C) MXene shows great potential for addressing challenging implant-associated infections in bone regeneration due to its strong photothermal conversion efficiency. However, its photothermal efficacy is restricted to the near-infrared I (NIR-I) region due to a limited absorption range. To address this, we designed platinum nanoparticle-decorated V_2_C heterostructures (Pt@V_2_C) via an in situ growth method, leveraging Pt’s plasmonic and catalytic properties to extend the photoresponse to the NIR-II window. Subsequently, Pt@V_2_C was integrated into poly-L-lactic acid (PLLA) to fabricate PLLA-Pt@V_2_C scaffolds with photothermal antibacterial function by selective laser sintering. The optimized PLLA-Pt@V_2_C scaffold achieves a record photothermal conversion efficiency (56.03% at 1064 nm), triggering simultaneous hyperthermia (>52 °C) and catalytic ·OH radical generation. In vitro studies demonstrate exceptional antibacterial efficacy against Staphylococcus aureus and Escherichia coli, achieving over 99% killing rates upon 1064 nm near-infrared irradiation. Furthermore, the scaffold demonstrated significant inhibition of biofilm formation, achieving over 90% reduction in biofilm biomass. Moreover, the scaffold demonstrated high cell viability, confirming its dual functionality of potent bactericidal activity and biocompatibility that supports tissue regeneration. This work provides a feasible strategy for combating implant-associated infections.

## 1. Introduction

The escalating incidence of implant-associated bacterial infections presents a substantial clinical challenge, profoundly impacting patient morbidity and healthcare expenditure [1]. According to accumulating epidemiological evidence, infections linked to medical implants account for an estimated 20–30% of all implant failures and trigger a complex cascade of inflammatory responses, often leading to chronic inflammation, implant loosening, and eventual failure [2,3]. These infections place a significant economic burden on global healthcare systems, with associated costs exceeding $3.5 billion annually as reported by the World Health Organization. Despite the increasing use of implant biomaterials for bone defect reconstruction, effective strategies for preventing and treating implant-related infections are still severely lacking [4,5]. Currently, antibiotics remain the primary strategy for combating bacterial infections [6,7]. However, the lack of vascularization in the bone defect area leads to rapid bacterial adhesion on the implant surface and subsequent biofilm formation [8]. This biofilm not only compromises the host’s immune defense but also severely diminishes the efficacy of traditional antibiotics, thereby significantly increasing the risk of implant failure [9]. The limitations of antibiotics against biofilms, the need for antibiotic-independent therapies, and the challenge of finding materials that can both control infection and support regeneration [10].

Photothermal therapy (PTT) has recently emerged as a promising avenue for addressing implant-associated infections, garnering significant attention due to its spatiotemporal precision, deep tissue penetration, and ability to circumvent the limitations of antibiotic resistance [11,12]. PTT employs photothermal agents (PTAs) to convert absorbed near-infrared (NIR) light into localized thermal energy, disrupting bacterial structures and leading to their inactivation. The development of highly efficient PTAs is therefore a pivotal focus for PTT research. Recent advances have positioned two-dimensional (2D) nanomaterials, such as V_2_C MXene, as compelling PTA candidates. V_2_C MXene exhibits exceptional photothermal conversion efficiency, achieving a value as high as 45.3% under 808 nm NIR light excitation. The inherent biocompatibility of V_2_C MXene further enhances its suitability for antibacterial applications, particularly in combating bacterial infections within bone regeneration [13]. However, several limitations persist. The primary challenge is that the reliance on NIR-I excitation (700–900 nm) and a singular photothermal mechanism restricts the capacity to overcome critical hurdles, including efficient biofilm penetration and the generation of sufficient oxidative stress for complete bacterial eradication [14]. To enhance therapeutic efficacy, it is crucial to explore alternative strategies, including the transition towards the NIR-II window (1000–1700 nm), which offers superior tissue penetration (up to 8 mm), reduced scattering losses and, consequently, improved therapeutic outcomes [15].

Furthermore, the incorporation of plasmonic nanomaterials provides a compelling strategy to enhance PTT efficacy and address some of these limitations. Among them, platinum nanoparticles (Pt NPs) are particularly attractive candidates due to their inherent catalytic activity and strong spectrum surface plasmon resonance (SPR) effect within the NIR-II region [16,17]. Under NIR-II irradiation, the SPR excitation of Pt NPs generates hot electrons, which transfer efficiently via non-radiative energy transfer to the V_2_C MXene. This process markedly boosts the carrier concentration within the MXene [18]. The resultant increase in charge carriers not only enhances the photothermal performance but is also expected to augment catalytic activity, collectively leading to superior antibacterial efficacy [19].

**Figure 1:**
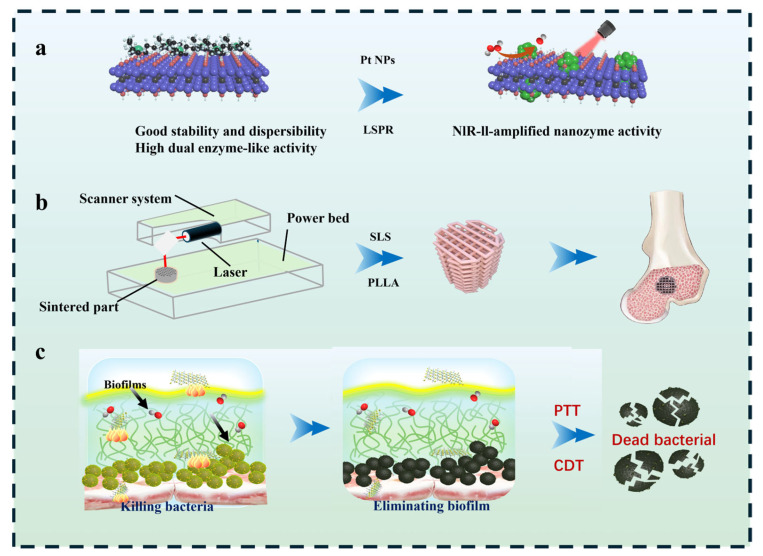
(**a**) Synthesis of the Pt@V_2_C nanoplatform and its schematic structure diagram. (**b**) Schematic illustration of the 3D-printed scaffold fabrication process. (**c**) Near-infrared II-enhanced nanozyme strategy for efficient eradication of methicillin-resistant Staphylococcus aureus and biofilm removal.

While studies have explored various antibacterial agents and polymer scaffolds separately, a significant challenge remains in developing integrated biomaterials that can effectively tackle established biofilm infections while simultaneously promoting the osteogenic process. In this study, a NIR-II light-activated antibacterial heterojunction was constructed by growing Pt NPs in situ on V_2_C MXene (Pt@V_2_C). This engineered Pt@V_2_C was then introduced into poly-L-lactic acid (PLLA) to prepare 3D antibacterial bone scaffolds via selective laser sintering technology [20]. This antibacterial scaffold integrates a potent photothermal agent with a biocompatible matrix, aiming to provide antimicrobial efficacy against bacteria relevant to infectious bone defects upon NIR-II irradiation. Further studies will rigorously evaluate its ability to suppress mature biofilm formation and assess potential eradication strategies (Figure 1).

## 2. Materials and Instruments

### 2.1. Materials

Poly(L-lactic acid) (PLLA; Mw ≈ 150 kDa) was obtained from Shenzhen Baolite Biomaterials Co., Ltd. (Shenzhen, China). V_2_C MXene nanosheets, chloroplatinic acid hexahydrate (H_2_PtCl_6_·6H_2_O), methylene blue (MB), 1,3-diphenylisobenzofuran (DPBF), dimethyl sulfoxide (DMSO), tryptone, agar powder, yeast extract, and sodium chloride (NaCl) were purchased from Aladdin Biochemical Technology Co., Ltd. (Shanghai, China).

### 2.2. Synthesis of Pt@V_2_C

Pt nanoparticles were loaded onto V_2_C MXene nanosheets via wet impregnation. Briefly, 100 mg of V_2_C was dispersed in 20 mL deionized water and sonicated to obtain a homogeneous suspension. Subsequently, an aqueous solution of H_2_PtCl_6_·6H_2_O (0.02 g/mL) was added dropwise under stirring [21]. The pH value was adjusted to 9 by slowly adding NaOH solution. After stirring for 30 min, an aqueous NaBH_4_ solution was added dropwise to reduce platinum ions, and the mixture was stirred for 12 h at room temperature [22,23]. The resulting precipitate was collected through centrifugation at 8000 rpm for 10 min, washed three times with ethanol and deionized water, and dried under vacuum for 12 h to yield Pt@V_2_C nanosheets [24].

### 2.3. Characterization of Pt@V_2_C

The morphology and elemental composition of Pt@V_2_C were characterized via transmission electron microscopy (TEM), selected area electron diffraction (SAED), and energy-dispersive X-ray spectroscopy (EDS), performed on a Thermo Fisher Talos F200S G2 (Waltham, MA, USA). The crystalline structure was analyzed by X-ray diffraction (XRD) using a D8 Advance (Bruker, Bremen, Germany). Electron paramagnetic resonance (EPR) spectra were recorded on a Bruker EMXplus-6/1 (Germany). The surface chemical states were examined through X-ray photoelectron spectroscopy (XPS) using a Thermo Scientific ESCALAB 250Xi (USA).

### 2.4. Preparation of the Composite Scaffolds

Selective laser sintering (SLS) was employed for scaffold fabrication due to its ability to precisely control pore size, architecture, and interconnectivity, crucial for optimal bone ingrowth and vascularization. This technique also ensures the homogeneous incorporation and stabilization of the Pt@V_2_C nanoparticles throughout the PLLA matrix, preventing aggregation and facilitating uniform activation. In detail, polylactic acid (PLLA) powder was mixed with V_2_C and Pt@V_2_C powders in ethanol and stirred at room temperature for 1 h using a magnetic stirrer. After centrifugation, drying, and grinding, PLLA-V_2_C and PLLA-Pt@V_2_C composite powders were obtained (Pt@V_2_C content 4 wt.%). The SLS process involved importing 3D model slicing data, spreading powder with a scraper, selectively sintering with a laser, and repeating powder spreading and sintering layer by layer until the scaffold was formed. Optimal printing parameters were laser power 3.6 W, scan spacing 0.10 mm, pulse width 8 μs, scanning speed 120 mm/s, and build platform maintained at 120 °C. Porous interconnected scaffolds were finally obtained, named PLLA, PLLA-V_2_C, and PLLA-Pt@V_2_C.

### 2.5. The Photothermal Performance Test

To test the photothermal performance of the scaffolds, each scaffold type was immersed in phosphate-buffered saline (PBS) within a 3 mL Eppendorf tube. The samples were irradiated with a 1064 nm continuous wave laser (power density: 1 W/cm^2^). Surface temperature changes were monitored using a FLIR E75 infrared thermal camera and a multiplexed temperature recorder (MT-8X, Shenhua Xuanke Technology Co., Ltd., China). The stability of photothermal performance over multiple irradiation cycles was evaluated through four on/off switching cycles. The photothermal conversion efficiency (*η*) was calculated using the method described in the previous literature.

(1) $$\eta =\frac{hs(T_{\max}-T_{sur})-Q_{DIS}}{I(1-10^{-A})}$$

### 2.6. The Chemodynamic Performance Test

·OH generation was quantified by TMB oxidation. Specifically, scaffolds of each group (PLLA, PLLA-V_2_C, PLLA-Pt@V_2_C) were immersed in PBS containing 3,3′,5,5′-tetramethylbenzidine (TMB, 0.5 mM) at a pH value of 5.5. After incubation at 37 °C for 10 min, UV-Vis absorption spectra were recorded at 370 nm at designated time points to determine ·OH production. Additionally, ESR spectroscopy with 5,5′-dimethyl-1-pyrrolidine-N-oxide (DMPO) as a spin trap was employed to detect ·OH radicals. Mix PLLA-Pt@V_2_C with the scaffold in PBS (pH 5.5) with DMPO (5 mM) and record its ESR spectrum.

### 2.7. Density Functional Theory Calculations

A V_2_C model and a Pt nanoparticle-loaded V_2_C model were constructed, each comprising three atomic layers. The DFT method within the VASP software (VASP 6.1) package was employed to investigate the geometric optimization, charge density differences, electronic band structures, and density of states of V_2_C@Pt.

### 2.8. Antibacterial Assay of Plla-Pt@V_2_C Scaffold In Vitro

#### 2.8.1. Spread Plate Method

The antibacterial performance of the scaffold was evaluated using the spread plate method with Gram-negative Escherichia coli (*E. coli*, ATCC 25923) and Gram-positive Staphylococcus aureus (*S. areus*, ATCC 25922). The bacterial culture medium contained 1% sodium chloride, 0.5% yeast extract, and 1% peptone. Prior to co-culture, scaffolds were sterilized by immersion in 75% ethanol for 30 min, followed by UV irradiation for an additional 30 min. Bacterial suspensions with an OD600 of 0.05 were then mixed with the scaffolds in 48-well plates and co-cultured at 37 °C in a constant-temperature incubator for 6 h. The scaffolds were then rinsed with PBS, and 1 mL of soluble broth medium was added and shaken for 10 min. Finally, the bacterial suspension was diluted 10^4^-fold, and 25 μL of the diluted suspension was evenly spread onto LB agar and incubated at 37 °C for 14 h. Post-incubation, the survival conditions of *E. coli* and *S. aureus* were recorded using a digital camera, and bacterial viability was assessed using the colony count method.

#### 2.8.2. Antibiofilm Assay In Vitro

Biofilm formation was evaluated via crystal violet staining to quantify the extent of biofilm biomass formed. Briefly, scaffolds in 48-well plates were incubated with bacterial suspensions (10^6^ CFU/mL) for 6 h at 37 °C. Subsequently, the scaffolds were irradiated with a 1064 nm laser (1.0 W/cm^2^, 10 min). After irradiation, the scaffolds were removed, and the remaining culture medium was incubated for an additional 48 h to promote biofilm formation. The biofilms were fixed with methanol, stained with 0.1% crystal violet for 30 min, washed thoroughly, and then dissolved with 33% acetic acid. The absorbance at 595 nm was measured to quantify the biofilm biomass.

#### 2.8.3. Bacterial Morphology Observations

To evaluate the antibacterial performance of the scaffold, the morphology of the bacteria on its surface was observed using a scanning electron microscope (SEM). Different scaffolds were co-cultured with a bacterial solution (10^6^ CFU/mL) in a 48-well plate and incubated in a constant-temperature incubator at 37 °C for six hours. The scaffolds were then irradiated with 1064 nm near-infrared (NIR) light for ten minutes. The scaffolds were fixed with 2.5% glutaraldehyde for 30 min before being dehydrated using a gradient of ethanol solutions (30%, 50%, 75%, 80%, 90%, 95%, and 100%). After thorough drying, the scaffolds were subjected to gold-sputtering treatment, and the bacterial morphology was subsequently observed using scanning electron microscopy.

#### 2.8.4. Intracellular Ros Detection

Bacteria were cultured on scaffolds in confocal dishes for 24 h, then subjected to 1064 nm laser irradiation (1.0 W/cm^2^, 10 min). After washing with PBS, the bacteria were stained with DCFH-DA (10 μM) for 30 min in the dark. Fluorescence images were captured using an Olympus BX51 fluorescence microscope to visualize intracellular ROS production in bacteria.

### 2.9. Cytocompatibility Evaluation

To assess cytotoxicity, L929 cells were co-cultured with PLLA, PLLA-V_2_C, and PLLA-Pt@V_2_C scaffolds. On days 1 and 3 of the culture period, a classic double-staining protocol was employed using calcein-AM (green) and propidium iodide (red), whereby live cells were stained green and dead cells red. The samples were observed under a fluorescence microscope (BX51, Olympus, Tokyo, Japan) at predetermined time points. Cell numbers were then detected using the Cell Counting Kit-8 (CCK-8, Solarbio, Beijing, China). The brief steps are as follows: After repeating the above co-culture and seeding steps, add α-MEM medium containing a 10% CCK-8 working solution to each well, then incubate at 37 °C in the dark for 1 h. Measure the absorbance at 450 nm using a microplate reader (Varioskan LUX, Thermo Scientific, USA).

### 2.10. Statistical Analysis

All quantitative data are expressed as means ± standard deviation (SD). Statistical significance between two groups was evaluated using Student’s *t*-test. Analyses were performed with Origin Pro 2023b.

## 3. Results and Discussion

### 3.1. Synthesis and Characterization of Pt@V_2_C

V_2_C MXene nanosheets were synthesized via etching. The synthesis process was followed by intercalation with tetramethylammonium hydroxide (TMAOH) and ultrasonication to produce nanosheets with large surface areas. These nanosheets possess excellent photothermal and catalytic properties, which facilitate the effective loading of platinum (Pt) nanoparticles. These Pt nanoparticles can decompose H_2_O_2_ or H_2_O into ·OH radicals, potentially enhancing sterilization efficacy through synergistic mechanisms.

**Figure 2:**
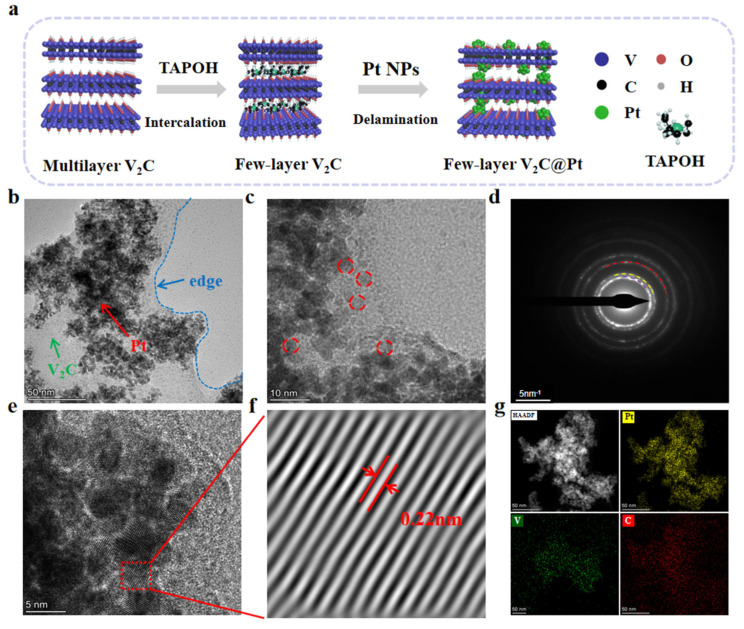
(**a**) Schematic diagram of the synthesis process. (**b**,**c**) TEM images. (**d**) SAED pattern of Pt@V_2_C. (**e**,**f**) High-resolution STEM images showing clear lattice fringing. (**g**) EDS analysis of Pt@V_2_C.

Using a simple wet impregnation reduction method, Pt nanoparticles were introduced to occupy the V sites, forming the Pt@V_2_C nano system (Figure 2a). Transmission electron microscopy (TEM) images revealed that few-layer V_2_C supports a large number of Pt NPs (Figure 2b). High-resolution TEM images indicated that the Pt nanoparticles deposited on the surface of V_2_C MXene had an average diameter of 3–5 nm (red dashed circles) (Figure 2c). Subsequent inverse Fourier transformation of selected regions confirmed the successful loading of Pt nanoparticles through analysis of the diffraction ring and the calculated 0.22 nm lattice spacing, corresponding to the Pt(111) crystal plane (Figure 2d,e). Furthermore, TEM elemental mapping analysis showed that carbon, vanadium, and platinum were uniformly distributed throughout the Pt@V_2_C composite (Figure 2g). These results demonstrate the successful synthesis of Pt@V_2_C.

**Figure 3:**
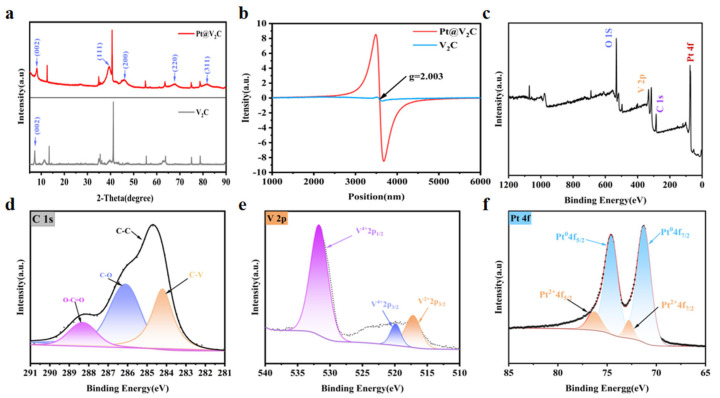
(**a**) Powder XRD patterns of V_2_C and Pt@V_2_C. (**b**) EPR spectra of Pt@V_2_C. (**c**) Pt@V_2_C XPS full survey. (**d**–**f**) Corresponding detailed regions of C (**d**), V (**e**) and Pt (**f**) of Pt@V_2_C by X-ray photoelectron spectroscopy (XPS) analysis.

X-ray diffraction (XRD) spectra were used to analyze the phase composition of the synthesized materials. The XRD pattern of V_2_C exhibited a characteristic (002) plane peak at approximately 9.1°, which is indicative of the interlayer spacing in delaminated MXene nanosheets and confirms the successful removal of the Al layer from the parent V_2_AlC MAX phase (Figure 3a). However, weak but discernible diffraction peaks corresponding to the unreacted V_2_AlC MAX phase were also observed, suggesting the presence of residual precursor material. The incomplete etching process, as evidenced by these residual peaks, may arise from kinetic limitations during the HF-based delamination step, particularly in regions where the Al layer is less accessible due to structural densification or local variations in intercalation efficiency [25]. In the XRD pattern of Pt@V_2_C nanosheets, diffraction peaks corresponding to both V_2_C MXene and Pt were observed. By comparison with standard PDF cards, these peaks were identified as representing the (111), (200), (220), and (311) planes of Pt [26]. A pair of peaks (g = 2.003) was observed in the electron paramagnetic resonance (EPR) spectra of V_2_C and Pt@V_2_C, indicating the presence of high-density defects in Pt@V_2_C (Figure 3b).

X-ray photoelectron spectroscopy (XPS) was employed to investigate the surface composition, properties, and electronic states of Pt@V_2_C. The full XPS spectrum showed peaks for V, O, C, and Pt (Figure 3c). The C 1s fine spectrum exhibited peaks at 284.21, 284.8, 286.11, and 288.31 eV, attributed to C-V, C-C, C-O, and O-C=O, respectively (Figure 3d). The V 2p XPS spectrum displayed peaks at 531.68 eV, 519.88 eV, and 517.18 eV, corresponding to V-O (V^4+^ 2p_1_/_2_), C-V (V^2+^ 2p_3_/_2_), and V-O (V^4+^ 2p_3_/_2_), respectively (Figure 3e). Notably, the Pt 4f XPS spectrum indicated that the peaks at 76.28 and 71.28 eV corresponded to Pt^2+^ and Pt^0^, respectively (Figure 3f). Collectively, these results confirm the successful synthesis of Pt@V_2_C and indicate a stable chemical interaction between Pt nanoparticles and V_2_C MXene. This interaction is essential for the envisioned synergistic catalytic and photothermal activities, potentially facilitating electron transfer and enhancing catalytic performance.

### 3.2. Photothermal Effect of Plla-Pt@V_2_c Scaffold

**Figure 4:**
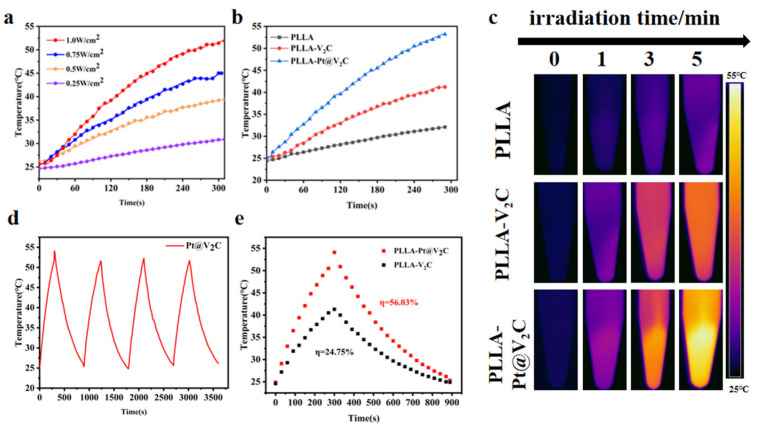
(**a**) Photothermal curves of PLLA-Pt@V_2_C at different powers. (**b**) Photothermal curves of different materials at the same time (1064 nm,1 W cm^2^). (**c**) Photostability of plla-V_2_C and PLLA-Pt@V_2_C under 1064 nm laser irradiation for four cycles (1.0 W cm^−2^). (**d**) Heating and cooling curves of PLLA-V_2_C and PLLA-Pt@V_2_C. (**e**) Infrared thermograms of different scaffolds irradiated with near-infrared light.

The photothermal performance of the PLLA-Pt@V_2_C composite scaffold was evaluated by monitoring temperature elevation under NIR-II laser irradiation with various power densities. We investigated the temperature rise curves of the PLLA-Pt@V_2_C composite material at different power levels [27]. As shown in Figure 4a, the temperature rise was directly proportional to the laser power density, reaching a maximum of 51.9 °C at 1 W/cm^2^. Therefore, the PLLA-Pt@V_2_C scaffold demonstrated a robust and efficient photothermal effect upon NIR-II laser irradiation.

Further investigation was conducted into the temperature differences of different scaffolds under the same illumination time. The temperature rise of the PLLA scaffold after laser irradiation was negligible, while both the PLLA-V_2_C and PLLA-Pt@V_2_C groups showed a temperature increase. The PLLA-V_2_C group only warmed up to 41.2 °C. In contrast, the PLLA-Pt@V_2_C group exhibited a significant temperature increase, indicating that Pt@V_2_C loaded with Pt has an excellent photothermal effect (Figure 4b). This enhancement underscores the critical role of platinum in boosting the photothermal conversion efficiency [28]. The stability of the photothermal effect was assessed via four consecutive on/off irradiation cycles, during which the PLLA-Pt@V_2_C scaffold maintained consistent temperature profiles (Figure 4d). Based on maximum steady-state temperatures and heat transfer dynamics, the photothermal conversion efficiency (PCE) was calculated to be 24.75% for V_2_C alone and 56.03% for Pt@V_2_C. These findings demonstrate that PLLA-Pt@V_2_C possesses advantageous properties for applications requiring rapid and stable thermal responses. This excellent photothermal property is a key attribute for various biomedical applications, including hyperthermia-based cancer therapy and antibacterial treatments.

### 3.3. Chemodynamic Effect of Plla-Pt@V2c Scaffold

**Figure 5:**
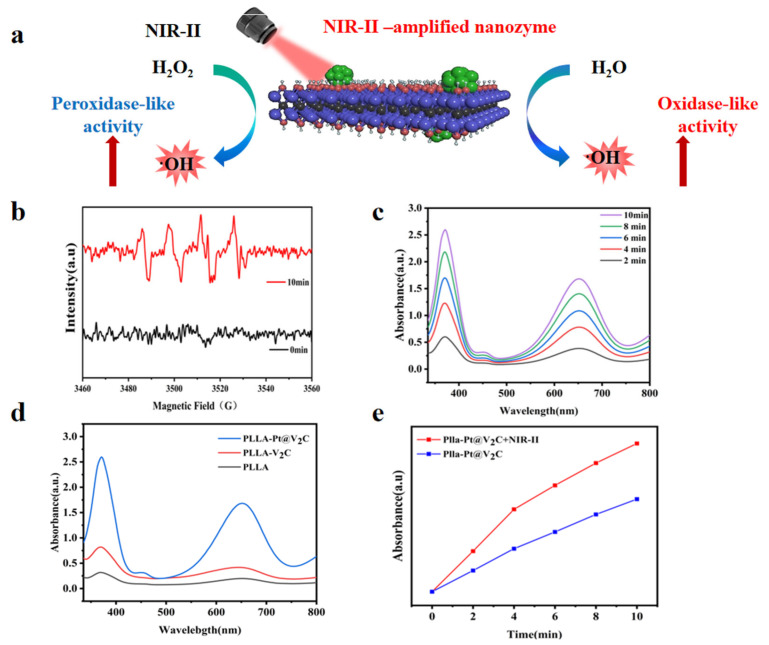
(**a**) Schematic representation of the NIR-II amplified enzyme-like activity of Pt@V_2_C. (**b**) ESR spectra of PLLA-Pt@V_2_C scaffolds under acidic conditions. (**c**) UV-Vis absorption spectra of different scaffolds probed with TMB under acidic conditions (pH = 5.5). (**d**) UV-visible absorption spectra of 3,3,5,5-tetramethylbenzidine (TMB) solution containing PLLA-Pt@V_2_C under different illumination times. (**e**) Comparison of PLLA-Pt@V_2_C group under illumination and without illumination.

The PLLA-Pt@V_2_C scaffold exhibits an excellent chemodynamic effect and under near-infrared light irradiation it can generate more reactive oxygen species (ROS). The ability of the scaffold to generate ·OH was verified by electron spin resonance (ESR) spectroscopy [29]. After 10 min of irradiation with a 1064 nm NIR laser, the characteristic ESR signal of hydroxyl radicals was observed (Figure 5b), providing direct evidence of ·OH radical generation.

To investigate the efficiency of ROS production, 3,3′,5,5′-tetramethylbenzidine (TMB) was used to detect the generation of ·OH. TMB is an ·OH scavenger that rapidly reacts with ·OH to form a product with an absorption peak near 652 nm. When the PLLA-Pt@V_2_C scaffold was added to the TMB mixture, the characteristic absorption of TMB increased steadily, indicating a reaction time dependence of the scaffold (Figure 5c). After co-culturing each group of scaffolds with TMB for 10 min, it was evident that the absorption change in pure PLLA was negligible. The PLLA-V_2_C group showed a slight increase, while the PLLA-Pt@V_2_C group exhibited a significant elevation in the absorption curve, with a notable rise in the characteristic absorption of TMB (Figure 5d). The enhanced ROS generation upon NIR-II laser irradiation (Figure 5e) suggests that the synergistic combination of the scaffold’s components and external light stimulation is crucial for maximizing the chemodynamic activity. This observation is consistent with the proposed mechanism where Pt NPs catalyze the decomposition of endogenous H_2_O_2_ into highly cytotoxic ·OH radicals. The observed strong chemodynamic effect of PLLA-Pt@V_2_C is vital for its antimicrobial applications, as ROS are known to inflict significant damage on bacterial cells.

### 3.4. Density Functional Theory Calculation of Pt@V_2_c

**Figure 6:**
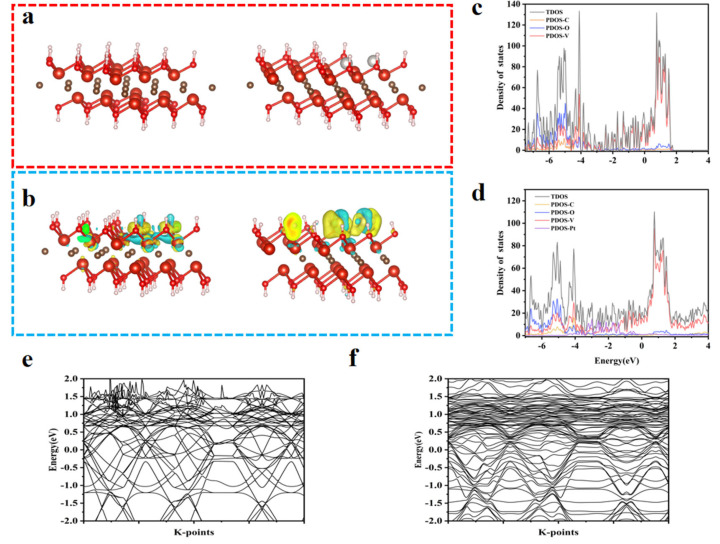
DFT theoretical calculations for V_2_C and Pt@V_2_C. (**a**) Optimized structure of Pt@V_2_C. (**b**) Charge density difference map. Density of states for (**c**) V_2_C and (**d**) Pt@V_2_C. (**e**) V_2_C and (**f**) Pt@ V_2_C bandgap between the valence band maximum (VBM) and conduction band minimum (CBM).

We further investigated the characteristics of Pt@V_2_C nanostructures based on density functional theory (DFT) calculations, thereby elucidating the mechanism underlying their biocatalytic (peroxidase, POD-like) activity [17,30]. The focus of this study was to analyze the interaction properties between Pt NPs and V_2_C, as well as their influence on the electronic structure. Figure 6a shows the optimized lattice structure and its spatial geometric distribution. The calculated electron density distribution (Figure 6b) reveals that V_2_C exhibits uniform electron dispersion, whereas Pt@V_2_C shows significant electron density aggregation at the Pt NP sites. This indicates that the Pt NP-loaded Pt@V_2_C system is in an activated state. Further density of states (DOS) analysis (Figure 6c,d) and band gap studies (Figure 6e,f) demonstrate that near the Fermi level of V_2_C, electrons are dominated by V 2p and C 2p orbitals. In contrast, the interaction between Pt 4f electrons and the Fermi level in the Pt@V_2_C system induces spin polarization.

These theoretical insights support the experimental findings regarding the catalytic potential of Pt@V_2_C, particularly its peroxidase-like activity in generating ROS. The enhanced catalytic performance is likely due to the modified electronic structure and increased surface reactivity of Pt NPs upon interaction with the V_2_C substrate.

### 3.5. In Vitro Antibacterial and Anti-Biofilm Efficacy

**Figure 7:**
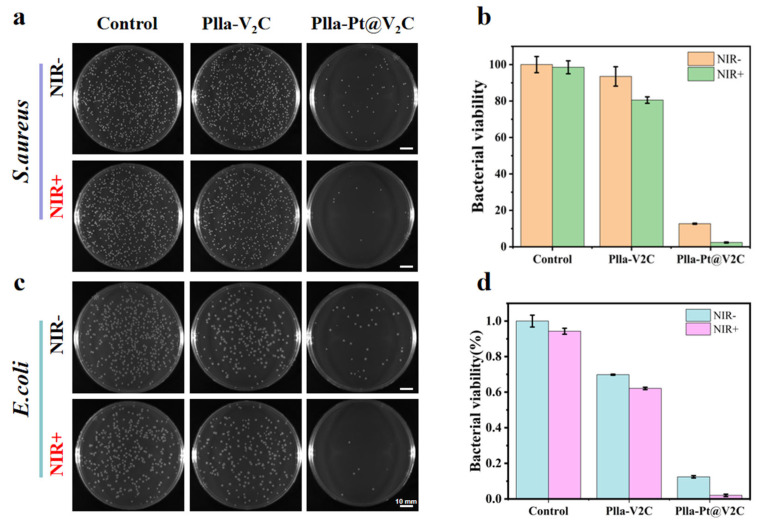
Antimicrobial testing: PLLA, PLLA-V_2_C, and PLLA-Pt@V_2_C treated in the dark or under 1064 nm laser (1.0 W/cm^2^ for 5 min) irradiation for (**a**) *S. aureus* panel results and (**b**) corresponding antimicrobial rates. Same conditions after treatment (**c**) *S. aureus* spread plate results and (**d**) corresponding antimicrobial rates (*n* = 3).

Delayed healing and severe inflammatory reactions caused by bacterial infections are prone to occur during tissue regeneration and repair [31]. While the development of antibiotic-free antibacterial strategies is of paramount importance, a crucial challenge lies in creating advanced biomaterial systems that can proactively inhibit biofilm formation while simultaneously promoting regenerative processes. This study presents a significant advancement by integrating the synergistic photothermal and chemodynamic antibacterial agent Pt@V_2_C nanoparticles into a PLLA scaffold fabricated via SLS technology [32]. This combination offers a novel, multifunctioning implant material designed to combat infection at its earliest stages. In the aforementioned experiments, we confirmed that PLLA-Pt@V_2_C exhibits excellent photothermal and chemodynamic effects. To further validate these results, we performed qualitative and quantitative analyses of different scaffold groups using the standard colony counting method for *S. aureus* and *E. coli*. *S. aureus* was mixed with each group of scaffolds and co-incubated. Under various experimental conditions, a substantial number of colonies were observed in both irradiated and non-irradiated PLLA groups. In contrast, a slight reduction in colony count was evident in the PLLA-V_2_C group; following near-infrared (NIR) light irradiation, the colony number decreased further due to the photothermal effect of V_2_C in the NIR-II region. Notably, the PLLA-Pt@V_2_C group without irradiation exhibited a significant reduction in colonies, while the PLLA-Pt@V_2_C + NIR-II group achieved over 90% bactericidal efficiency, confirming the synergistic antibacterial effect of chemodynamic therapy (CDT) and PTT (Figure 7a,b). Furthermore, we verified the antibacterial efficacy of PLLA-Pt@V_2_C against *E. coli* through additional experiments, which yielded consistent results. This demonstrates the scaffold’s capability to effectively neutralize free bacteria, a prerequisite for preventing biofilm establishment.

The above experiments demonstrate the satisfactory antibacterial effects of PLLA-Pt@V_2_C scaffolds as photothermal agents and nanoenzyme formulations [33]. Due to the protective nature of extracellular polymers, combating established biofilm-associated infections, particularly those caused by drug-resistant bacteria, is a complex task [34]. Inspired by the promising bactericidal properties of the PLLA-Pt@V_2_C scaffold, we conducted crystal violet staining experiments to evaluate its ability to inhibit the formation of biofilms of both *S. aureus* and *E. coli*. PLLA, PLLA-V_2_C, and PLLA-Pt@V_2_C scaffolds were co-cultured with *S. aureus* and exposed to NIR-II irradiation. The PLLA-V_2_C group exhibited a reduced rate of biofilm formation/increased biofilm inhibition. Notably, PLLA-Pt@V_2_C significantly suppressed biofilm formation and reduced the overall biofilm biomass through the synergistic action of CDT and more efficient PTT, achieving a significant effect on biofilm inhibition (Figure 7a). Subsequently, after dissolution in glacial acetic acid, the absorbance (OD) value at 595 nm, representing the quantified biofilm biomass, was measured using a microplate reader, yielding the corresponding trend (Figure 7b). Similar results were obtained in experiments with *E. coli*, demonstrating significant inhibition of biofilm formation (Figure 7c). These findings highlight the potential of PLLA-Pt@V_2_C scaffold for efficient sterilization and significant inhibition of biofilm formation, a critical step in preventing implant-associated infections. The synergy achieved through our tailored Pt@V_2_C integration within the SLS PLLA scaffold leads to enhanced NIR-II-promoted enzymatic activity and potent inhibition of biofilm formation, thereby improving its antibacterial efficacy beyond simple combinations of existing agents.

**Figure 8:**
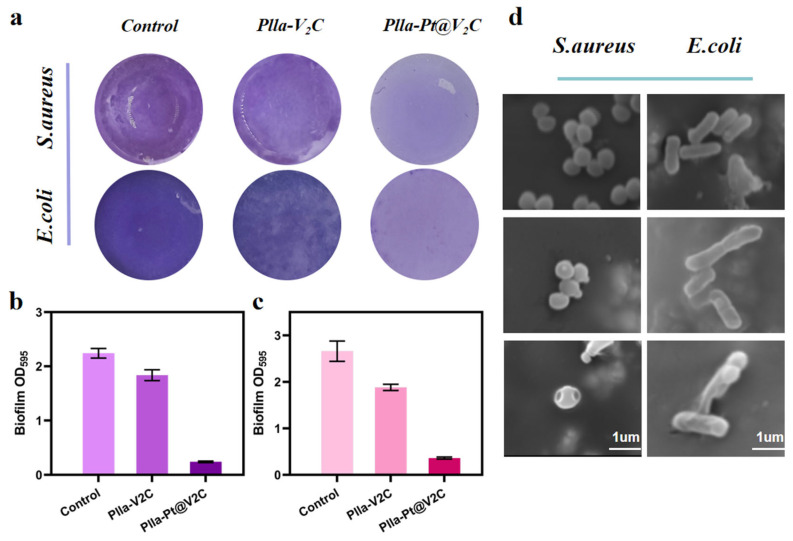
(**a**) Crystal violet staining analysis to quantify biofilm biomass after treatments aimed at inhibiting biofilm formation. (**b**,**c**) Relative biofilm biomass after treatments. Bacterial biofilm biomass was assessed at OD_595_ using an acetic acid system. (**d**) Scanning electron microscopy images showcasing the effect of treatments on bacterial morphology of *E. coli* and *S. aureus* on PLLA, PLLA-V_2_C, and PLLA-V_2_C@Pt scaffolds under 1064 nm laser irradiation following standard incubation periods.

We then investigated the antibacterial mechanism of Pt@V_2_C by examining the morphological changes in *S. aureus* and *E. coli*. Using scanning electron microscopy (SEM), we observed the microscopic morphology and structural integrity of the bacteria [35]. As shown in Figure 8d, the surface of the PLLA scaffold was covered with intact, smooth, spherical *S. aureus* and rod-shaped *E. coli.* The PLLA-V_2_C group exhibited moderate antibacterial activity: after NIR-II irradiation, the bacteria showed slight deformation, with cytoplasmic leakage and cell membrane wrinkling [36]. In contrast, the PLLA-Pt@V_2_C scaffold group induced severe bacterial membrane damage and deformation, indicating complete bacterial inactivation. This phenomenon is mainly attributed to the synergistic effects of reactive oxygen species (ROS) generated by the CDT of Pt@V_2_C and PTT-induced local hyperthermia. The combined effects increased the permeability of the bacterial membrane, thereby inactivating essential components such as proteins and lipids.

### 3.6. In Vitro Cytocompatibility

**Figure 9:**
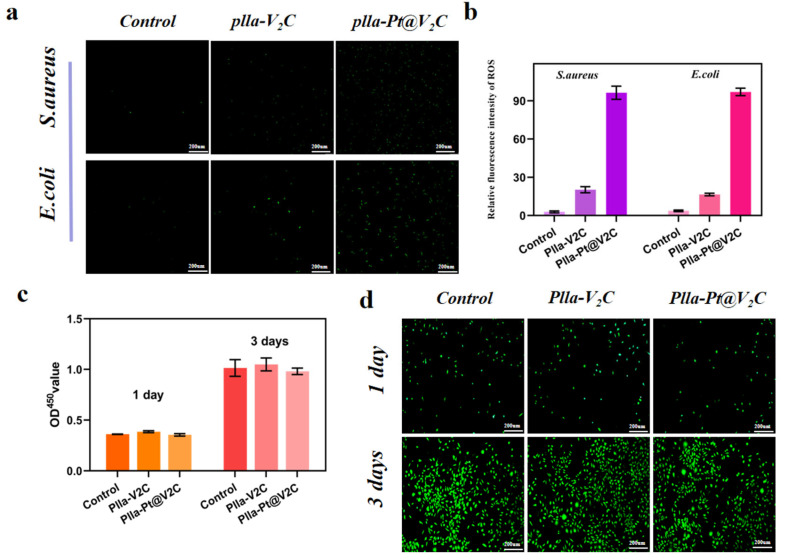
(**a**) The live/dead fluorescence staining images of mBMSCs co-cultured with different extracts, (**b**) the images of adhesion morphology on the different scaffolds for 3 days, and (**c**) the CCK-8 results and the viability rates of PLLA, PLLA-V_2_C and PLLA-V_2_C@Pt after 1 and 3 days’ culture. (**d**) Fluorescence images of live/dead staining of stem cells co-cultured with different extracts.

The ability of PLLA-Pt@V_2_C to generate ROS was further investigated at the cellular level using 2′,7′-dichlorofluorescein diacetate (DCFH-DA) [37]. This compound can be deacetylated by intracellular esterases and then converted into non-fluorescent DCFH [38]. DCFH emits green fluorescence when oxidized by ROS. We evaluated the ROS-generating ability of PLLA-Pt@V_2_C. Images of the different groups following treatment with a mixture of *S. aureus* and *E. coli* are presented in Figure 9a, showing the same trend as previous results. No distinct green fluorescence was observed in the PLLA group, while the PLLA-V_2_C group displayed merely a minimal amount of green fluorescence. The PLLA-Pt@V_2_C group exhibited the strongest green fluorescence, primarily due to the Pt NPs providing the composite scaffold with effective CDT performance and generating a significant quantity of ROS through the highly efficient photothermal effect [39].

The above experiments verified the excellent antibacterial performance of the scaffold materials. Meanwhile, to emphasize their good biocompatibility as bioengineered implant scaffolds, the effects of different scaffold materials on cell proliferation were evaluated by detecting the viability of mBMSCs using live/dead cell staining after co-cultivation with each scaffold for 1 and 3 days [40]. The results showed that cells in all groups exhibited good viability on both day 1 and day 3. Fluorescence microscopy images revealed abundant green fluorescence in all cell groups during the culture period, indicating a favorable growth trend with no significant accumulation of dead cells (Figure 9c). In addition, the CCK-8 was employed to assess the proliferation of mBMSCs co-cultured with each group of scaffold materials for 1 and 3 days. The experimental results demonstrated that all groups maintained stable cell growth, and no significant differences in cell proliferation levels were observed among the groups on day 3, which indicated that PLLA-Pt@V_2_C possesses favorable biocompatibility. These findings highlight the selective antibacterial nature of our Pt@V_2_C-enhanced CDT/PTT system. Such biocompatibility serves as a critical prerequisite for potential applications in regenerative medicine and implantable devices. The inherent biocompatibility of PLLA, combined with the SLS fabrication methods, further underscores the material’s potential for dual functionality in infection control and tissue regeneration [41,42]. The ability to achieve potent antibacterial effects while maintaining good cytocompatibility makes the PLLA-Pt@V_2_C scaffold a promising candidate for tissue engineering applications in infection-prone environments.

## 4. Conclusions

This study successfully synthesized Pt-decorated V_2_C MXene nanosheets via a simple wet impregnation–reduction method and integrated them into PLLA scaffolds through selective laser sintering. The resulting PLLA-Pt@V_2_C composite exhibited multifunctional properties, including efficient photothermal conversion, ROS generation, and chemodynamic catalytic activity. In vitro experiments demonstrated robust antibacterial efficacy and significant inhibition of biofilm formation, attributed to the synergistic PTT and CDT mechanisms activated under NIR-II irradiation. Additionally, the scaffold showed excellent biocompatibility with mammalian stem cells, highlighting its promise as a safe and effective platform for preventing and treating bacterial infections associated with bone implants. These findings pave the way for the development of advanced antibacterial biomaterials that harness nanotechnology-enabled multifunctionality for clinical translation.

## Data Availability

The original contributions presented in this study are included in the article. Further inquiries can be directed to the corresponding authors.
